# Décision d'amputation dans la prise en charge initiale d'une luxation ouverte grave de la cheville à propos d'un cas observé à l'Hôpital Laquintinie de Douala suite à un accident par moto-taxi

**Published:** 2012-12-05

**Authors:** Faustin Atemkeng Tsatedem, Jean Gustave Tsiagadigui, Richard Polle Ndando, Mohamadou Saidou Arabo, Alphonse Bayiha, Bruno Kenfack

**Affiliations:** 1 Département des Sciences Biomédicales, Faculté des Sciences Université de Dschang, Cameroun; 2 Faculté de Médecine et des Sciences Pharmaceutiques de l'Université de Douala, Cameroun; 3 Hôpital Laquintinie de Douala, Service d'orthopédie traumatologie, Cameroun

**Keywords:** Cheville, luxation ouverte, amputation, traumatisme, ankle, open dislocation, amputation, trauma

## Abstract

La décision d'amputation pour traumatisme grave de membre n'est pas toujours facile à prendre. Les auteurs rapportent le cas d'un traumatisme ouvert de la cheville gauche avec luxation tibiotalienne complète, référé pour amputation. Il s'agit d'une passagère d'une moto-taxi percutée par une voiture. A l'admission, le pouls tibial postérieur était présent et le score dit MESS (Mangled Extremity Severity Score) côté à 5, ce qui a permis et d'éviter l'amputation. Après débridement et réduction, une broche transplantaire a permis d'immobiliser la cheville et de faire les pansements. L'amputation a été évitée. La cicatrisation dirigée de la peau a été suivie par la kinésithérapie. La mobilité de la cheville autorise une marche avec cannes au quatrième mois post-opératoire. Les auteurs recommandent l'utilisation du MESS dans la décision d'amputation après traumatisme grave de membre.

## Introduction

L'augmentation du nombre de motos-taxis au Cameroun, l'excès de vitesse et le non respect du code de la route entre autres facteurs, entrainent l'accroissement des lésions traumatiques. La jambe, la cheville et le pied sont particulièrement exposés sur ces engins, et sont souvent l'objet de traumatismes graves. Ces traumatismes graves exposent au risque d'amputation, comme dans le cas clinique ici présenté.

## Patient et observation

Les auteurs rapportent le cas d'un traumatisme ouvert de la cheville gauche avec luxation tibiotalienne complète, référé pour amputation. Madame I.L.F., est une étudiante âgée de 25 ans, référée par un chirurgien de l'Hôpital Régional d'Edéa (HRE) à l'Hôpital Laquintinie de Douala (HLD) pour une luxation ouverte grave de la cheville gauche, après qu'elle ait refusé l'amputation qui lui a été proposée.

Elle était passagère d'une moto qui est entré en collision à Edéa sur l'axe lourd Douala-Yaoundé, avec une voiture roulant à vive allure, le 17 Septembre 2011 à 13H. Il s'ensuit un choc direct sur sa cheville gauche et une chute du même côté. Elle n'a pas perdu connaissance, a une large plaie et une importante déformation de sa cheville gauche associée à une impotence fonctionnelle immédiate. Les témoins l'amènent de toute urgence à l'HRE où un pansement compressif, un garrot, des antibiotiques antalgiques et du sérum antitétanique sont faits et un géloplasma perfusé avant sa référence à l'HLD. A l'admission, l'état général est moyen, la pression artérielle à 130/60mmHg, le pouls à 80 pulsations/minute, la fréquence respiratoire à 22 cycles par minute et le score de Glasgow à 15/15.

**Figure 1:**
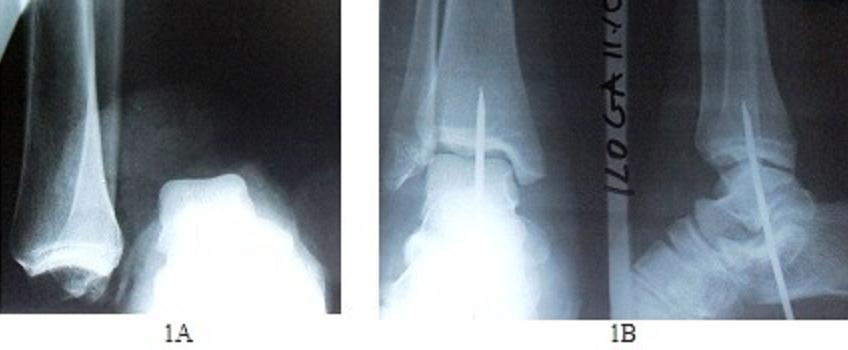
Radiographies (1A Préopératoire; 1B Postopératoire)

Localement, on note le garrot, le pansement compressif et une légère mobilité distale. La sensibilité plantaire est présente. Après avoir défait le garrot qui a duré une heure 10minutes, le pouls pédieux est absent mais le pouls tibial postérieur est présent mais faible. La cheville est très déformée. La radiographie montre une luxation tibio-talienne avec perte de contact total entre la mortaise tibiofibulaire et la poulie astragalienne et sans fracture (Figure 1A).

**Figure 2:**
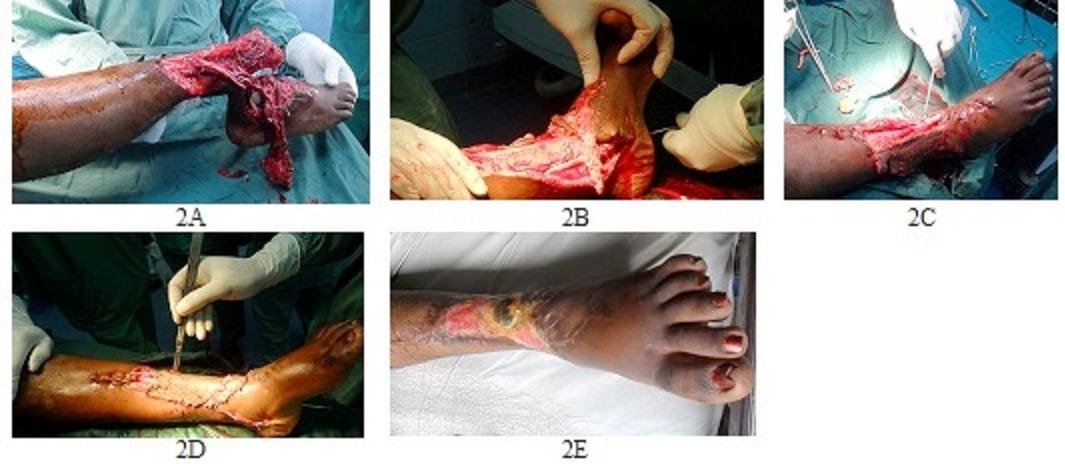
Aspect clinique (2A: lésions; 2B: Réduction et embrochage transplantaire; 2C: Rapprochement de la peau; 2D: Incisions de décharge; 2E: Aspect local à 4 mois postopératoire

Elle est opérée quatre heures après son traumatisme, sous anesthésie générale avec intubation orotrachéale. L'exploration montre (Figure 2A) une plaie articulaire de 10 cm de large, et 30 cm de long remontant vers la jambe, exposant le tibia, le péroné, la cheville, et des tendons fléchisseurs sectionnés de même que l'artère tibiale antérieure et les 2 veines saphènes. L'artère tibiale postérieure et ses deux veines satellites sont conservées avec un bon pouls après la réduction. Les deux branches terminales du nerf sciatique poplité externe que sont le musculocutané et le tibial antérieur sont également lésés. Le nerf tibial postérieur, branche terminale du nerf sciatique poplité interne est conservé. La syndesmose tibiofibulaire est conservée. La capsule articulaire est désinsérée du squelette jambier, de même que les ligaments latéraux. La peau est dévitalisée et rabattue en médial. Le score de prédiction MESS (Mangled Extremity Severity Score) est estimé à 5 (Tableau 1) et nous décidons de ne pas amputer. Nous réalisons un débridement, un lavage au salé, une réduction, un enclouage transplantaire (Figure 2B) à l'aide d'une broche de Steinman, une capsuloraphie, une réparation nerveuse, une ligamentoplastie, un rapprochement de la peau (Figure 2C), des incisions de décharge (Figure 2D), un pansement et une attèle plâtrée postérieure anti-équin.

**Tableau 1:** Score de sévérité des mutilations des extrémités (MESS). Amputation si total des points supérieur à 7. Pour notre patiente (en gras 4 + 1 = 5)

Groupes	Caractéristiques	Lésions	Points
Ischémie (points X2 si ischémie >6heures)	Légère	Pouls faible mais sans ischémie	+1
	Modérée	Pas de pouls, paresthésie, temps de recoloration cutané allongé	+2
	Avancée	Froideur, paralysie, anesthésie	+3
Age	< 30 ans		0
	30-50 ans		+1
	>50 ans		+2
Choc	Tension normale	Pression artérielle stable	0
	**Hypotension transitoire**	**Pression artérielle instable mais répondant aux perfusions intraveineuses sur le terrain**	**+1**
	Hypotension prolongée	Pression systolique < 90 mmHg, correction uniquement en réanimation	+2
Type de mécanisme lésionnel	Basse énergie	Contusion, fracture fermée	+1
	Moyenne énergie	Fractures ouvertes ou multiples luxations	+2
	Haute énergie	Ecrasement, arme à feu	+3
	**Très haute énergie**	**Avulsion tissulaire, contamination + +**	**+4**

Les suites opératoires sont simples. Le pouls tibial postérieur reste bien perceptible et la coloration du pied normale malgré l'oedème. La radiographie de contrôle postopératoire est satisfaisante (Figure 1B). Les soins locaux sont néanmoins prolongés, car il ya eu nécrose de la peau dévitalisée. L'ablation de la broche transplantaire a été faite à la huitième semaine. La greffe de peau prévue n'a pas été faite et la cicatrisation dirigée est obtenue au quatrième mois postopératoire (Figure 2E). La patiente marche après des séances de kinésithérapie, avec deux cannes et sans douleurs, au quatrième mois postopératoire.

## Discussion

Les luxations pures de la chevilles sont exceptionnelles [1]. Selon Soyer [2] seuls 73 cas ont été publiés dans la littérature. Le mécanisme lésionnel [3] implique une flexion plantaire forcée du pied qui déchire la capsule, les faisceaux antérieurs des ligaments latéraux et le rétinaculum des fléchisseurs. La compression axiale du tibia et le triceps attirent l'astragale en arrière. Dans notre cas la vitesse de l'agent vulnérant est incriminée. L'ouverture cutanée est fréquente et serait de l'ordre de 50% d'après Biga [4]. Les lésions fermées sont de bon pronostic après traitement orthopédique et plâtre pour 6 semaines. Les lésions ouvertes sont de pronostic réservé: chondropathie traumatique, lésions capsulo-ligamentaires et vasculo-nerveuses, pouvant imposer une arthrodèse, voire une amputation. La fixation externe est recommandée dans les fracas ouverts [4], mais un fixateur externe stérile n'est pas toujours disponible dans notre contexte, raison pour laquelle nous avons utilisé une broche de Steinman. L'amputation n'est à envisager en urgence qu'en cas d'écrasement ou de score dit MESS [5] supérieur ou égal à 7. Notre patiente était à 5, nous permettant de prendre la bonne décision (Tableau I). Nous reconnaissons toutes fois, que le pronostic à long termes de notre patiente, en termes d'arthrose précoce, de nécrose de l'astragale par exemple reste à évaluer à distance comme le souligne la littérature [4]. Cette lésion aurait pu être prévenue par la limitation de vitesse et une meilleure vigilance du moto-taximan.

## Conclusion

Le score dit MESS (Mangled Extremity Severity Score) est à recommandé dans des cas similaires.
